# Challenges in conducting genome-wide association studies in highly admixed multi-ethnic populations: the Generation R Study

**DOI:** 10.1007/s10654-015-9998-4

**Published:** 2015-03-12

**Authors:** Carolina Medina-Gomez, Janine Frédérique Felix, Karol Estrada, Marjoline Josephine Peters, Lizbeth Herrera, Claudia Jeanette Kruithof, Liesbeth Duijts, Albert Hofman, Cornelia Marja van Duijn, Andreas Gerardus Uitterlinden, Vincent Wilfred Vishal Jaddoe, Fernando Rivadeneira

**Affiliations:** 1 The Generation R Study Group, Erasmus University Medical Center, Rotterdam, The Netherlands; 2 Department of Epidemiology, Erasmus University Medical Center, Rotterdam, The Netherlands; 3 Department of Internal Medicine, Erasmus University Medical Center, Rotterdam, The Netherlands; 4 Analytic and Translational Genetics Unit, Department of Medicine, Massachusetts General Hospital, Harvard Medical School, Boston, MA USA; 5 Program in Medical and Population Genetics, Broad Institute, Cambridge, MD USA; 6 Division of Respiratory Medicine, Department of Pediatrics, Erasmus University Medical Center, Rotterdam, The Netherlands; 7 Division of Neonatology, Department of Pediatrics, Erasmus University Medical Center, Rotterdam, The Netherlands; 8 Department of Pediatrics, Erasmus University Medical Center, Rotterdam, The Netherlands; 9 Genetic Laboratory - Room Ee5-59b, Department of Internal Medicine, Erasmus University Medical Center, PO Box 2040, 3000 DR Rotterdam, The Netherlands

**Keywords:** Genome-wide association studies, Quality control GWAS, Genomic imputation, Admixed population, Multiethnic study

## Abstract

Genome-wide association studies (GWAS) have been successful in identifying loci associated with a wide range of complex human traits and diseases. Up to now, the majority of GWAS have focused on European populations. However, the inclusion of other ethnic groups as well as admixed populations in GWAS studies is rapidly rising following the pressing need to extrapolate findings to non-European populations and to increase statistical power. In this paper, we describe the methodological steps surrounding genetic data generation, quality control, study design and analytical procedures needed to run GWAS in the multiethnic and highly admixed Generation R Study, a large prospective birth cohort in Rotterdam, the Netherlands. Furthermore, we highlight a number of practical considerations and alternatives pertinent to the quality control and analysis of admixed GWAS data.

**Electronic supplementary material:**

The online version of this article (doi:10.1007/s10654-015-9998-4) contains supplementary material, which is available to authorized users.

## Introduction

Genome-wide association studies (GWAS) analyze a large number of single nucleotide polymorphism (SNPs) across the genome in a large number of samples, aiming to identify loci associated with complex traits at the population level. Since 2007, well-designed studies have been able to comprehensively test common genetic variation across the genome [1]. Up to now, at least 11,680 SNPs have been robustly associated with one or more complex traits, providing biological insight in traits as different as Alzheimer’s disease, prostate cancer, inflammatory bowel disease, obesity, stroke, diabetes, asthma, height, cholesterol levels and bone mineral density, to name only few examples of the successful performance of this approach [2].

Variants discovered by GWAS typically have small effects which is why minor sources of systematic or random error can lead to false positive associations or can mask real effects (false negative associations). In order to avoid bias, it is necessary to closely control the processes underlying the production of GWAS data, extending from laboratory processes (data generation) to imputation. It is also necessary to conduct statistical analyses, which incorporate factors into the models known to influence the trait of interest, as well as being appropriate to the characteristics of the study design.

Ethnicity is a confounder of epidemiological studies which incorporates cultural, geographical and biological dimensions. In the GWAS context spurious associations between genetic variants and a trait of interest occur when both allele frequencies and differences in trait distributions (disease prevalence or magnitude of quantitative traits), vary across ethnicities. From this perspective, adequate correction for potential population stratification is required for successful identification of genetics determinants of complex traits and diseases.

To date, GWAS have mainly focused on populations of European ancestry. Consequently, having another ethnic background is a common reason for exclusion of GWAS samples. As an illustration, from the 1734 GWAS papers indexed in the GWAS catalogue, 66 % included only individuals from European ancestry, 34 % included Non-Europeans only (most of those carried out in Asian populations), and 12 % included both Europeans and Non-European individuals [3]. Moreover, big consortia efforts such as Cohorts for Heart and Aging Research (CHARGE) or the Genetic Investigation of Anthropometric Traits (GIANT) have focused primarily on European populations, while efforts driven in populations of diverse ethnic background are of modest sample sizes. However, the inclusion of multiethnic and/or admixed populations in the analysis of GWAS can actually result in additional power. Firstly, larger datasets (representing higher power) can be assembled when the ancestry criterion is not used for sample exclusion. Keeping such “ethnic outliers” in the study also represents a better use of resources considering the logistic and burden behind sample collection and genotyping, and their associated costs. Secondly, the European-only approach has little power to detect association for genetic variants segregating at low frequency in European populations and statistical power can be gained if those variants are more common in other ancestries included in the analysis [4]. Some examples are provided by Fu et al., who describe variants associated with type 2 diabetes mapping to *UBE2E2* and *KCNQ1* that have higher frequencies in East Asians [minor allele frequency (MAF) of 0.22 and 0.38, respectively] as compared with Europeans (MAF of 0.093 and 0.08, respectively) [5]. Similarly, Wu et al. showed examples of ethnic specificity in variants associated with lipid levels mapping to *APOA5* and *APOB*. These very rare variants identified in African-Americans were not detected in either East Asian or European populations [6]. Further, (rare) variants specific to a subpopulation (e.g. a diabetes susceptibility variant arising in Native-Americans) can be identified in a derived highly admixed population (i.e. Mexicans) as having the largest effect [7]. In addition, population admixture, due to interbreeding of individuals from different origins, would have brought together genomes from continental populations, which are a product of genetic drift and different selective pressures. Following this line of reasoning, it is expected that recently admixed populations are likely to harbor a larger number of genetic variants than the original populations they come from [8]. Theoretically, this will result in a higher yield in the discovery of genetic determinants of complex traits. Another important genetic approach, suitable in admixed population, to identify disease risk variants is admixture mapping, which is powerful when the ancestral populations differ both in allele frequencies and disease prevalence. Then, in the vicinity of a disease locus, an affected individual should have a higher proportion of alleles inherited from the most affected ancestral population [9].

As GWAS worldwide are expanding to include multi-ethnic and admixed populations, we describe here the steps used for genetic data generation, study design and analytical procedures applied in the Generation R Study. The Generation R Study is a population-based prospective cohort following children and their mothers from fetal life onwards, which comprises a multiethnic population, including a high proportion of highly admixed individuals. We here mainly focus on how this approach can allow analyzing the whole set of individuals independent of genetic background as a mean to increase sample size and power to identify loci underlying complex traits and diseases. The different considerations described here can be applied to other multiethnic studies, particularly those of admix nature such as Hispanics or African Americans.

## Methods

### Study population

The Generation R Study is a multi-ethnic population-based prospective cohort study, spanning from fetal life until young adulthood, designed to identify early environmental and genetic causes of normal and abnormal growth, development and health during fetal life, childhood and young adulthood. Study design, data collection in prenatal and postnatal phases, and ethical issues of this study have been previously described in detail [10]. The Generation R Study is conducted in Rotterdam, the Netherlands, within a multi-cultural metropolitan area. The study area includes inhabitants of approximately 150 different ethnicities [11]. Pregnant women with a delivery date between April 2002 and January 2006 were informed about the study and provided written informed consent through their prenatal care provider during their first visit of the pregnancy. The medical ethics committee of Erasmus University Medical Center approved the study. In total, 9778 mothers were enrolled in the study.

The ethnic background of the children was defined by the parents’ country of birth, which was obtained by questionnaire. The participating child was defined as of non-Dutch ethnic origin if one of her/his parents was born abroad, and further classified using a socio-demographic definition as described by Statistics Netherlands [11]. If both parents were born abroad, the country of birth of the mother decided the classification of the ethnic background of the child. The ethnic background of the mother and partner were obtained in the same manner, based on their parents’ (the child’s grandparents) country of birth.

### Sample collection, biobanking and genotyping

Blood samples of the children were collected from the umbilical cord at birth. Where an umbilical cord blood sample could not be collected at birth, a blood sample was obtained by venipuncture during the child’s visit to the research center at a mean age of 6 years. All samples were coded with a unique laboratory number. Umbilical cord samples were collected in 10 ml EDTA tubes and stored immediately at −80 °C, while samples obtained by venipuncture were collected in 5 ml EDTA tubes and stored directly after transport at −20 °C. DNA was extracted manually from white blood cells using the Qiagen FlexiGene Kit (Qiagen Hilden, Germany). Normalisation and further processing of the DNA samples were performed on a Caliper ALH3000 pipetting robot. A detailed description of the Generation R Biobank has been previously published [12].

Genotyping was performed using Illumina HumanHap 610 or 660 Quad chips—depending on collection time—following manufacturer protocols, and intensities were obtained from the BeadArray Reader. Genotype calling was performed on normalized intensities using the Genecall module from the Illumina Genome Studio software version 1.1.0.28426. A no-call threshold of 0.15 was applied to a manufacturer-provided cluster file. Illumina Genome Studio provides a quality metric used to identify low-quality samples and, we used a threshold of 97.5 % for exclusion of samples.

### DNA quality control (QC)

The two Genome Studio projects (one each for the HumanHap 610 array and for the 660 array), were merged using SNPs common to both arrays. The QC procedures were applied to the genotyped data using PLINK [13] in two phases: marker- and sample-based.

**Fig. 1:**
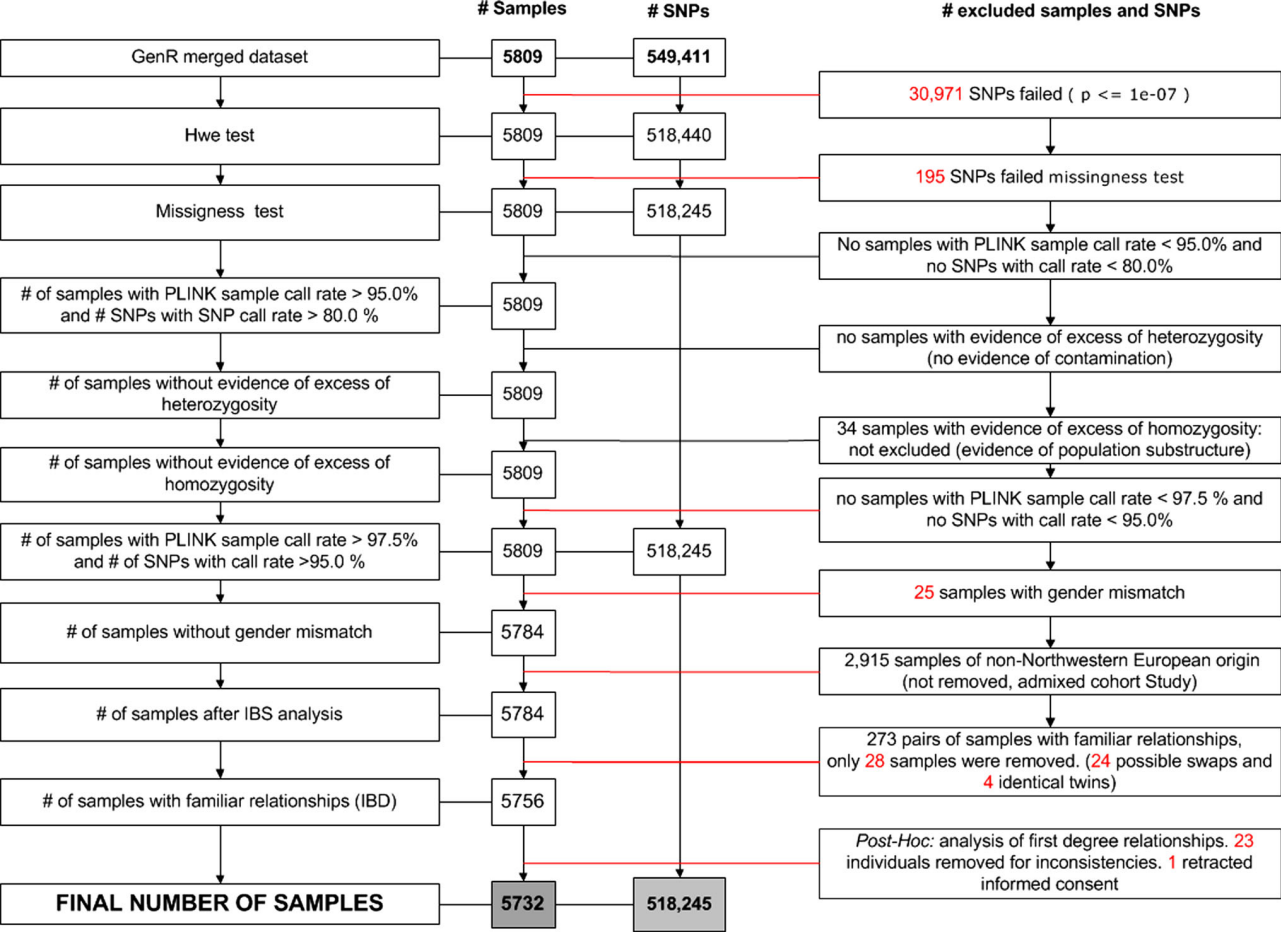
Flowchart overview of the entire GWAS QC process. Quality control of all samples from Generation R-1 and Generation R-2 after merging of the projects. *Red font* denotes exclusion of either SNPs or samples from the dataset in the different QC steps. (Color figure online)

Marker QC included filters for: (1) marker call rate (calling <0.2 – <0.05, --geno option), checked in two rounds, the initial with a threshold of 80 % and the second one more stringent (95 %), after inspection of sample quality, (2) minor allele frequency (MAF ≤ 0.001, --maf option), (3) differential missingness between the two projects (*P* < 1 × 10^−7^, --test-missing option) and (4) deviation from Hardy–Weinberg equilibrium proportion (*P* < 10^−7^
--hwe option). Sample QC included: (1) duplicate detection (PLINK option IBS = 1), (2) sex discordance rates (--check-sex option), comparing the reported sex of each participant with the sex predicted by the genetic data (expected chromosome X heterozygosity). When results were inconclusive, the Genome Studio plots, log R ratios and B-allele frequencies, for both X and Y chromosomes were inspected. (3) Genotype call rate (<0.05 – <0.025--mind option) checked in two rounds, the initial with a threshold of 95 % and the second one more stringent (97.5 %), after inspection of marker quality and (4) high heterozygosity rate, over 4 SD of the mean heterozygosity of all samples (--het option). The step by step summary of the applied QC pipeline is presented in Fig. 1, and Online Resources 1 and 2.

### Population sub-structure and family relationships

Additional sample QC assessments were applied to determine genetic-based ethnic background and to identify potential family relationships.

#### Genetic ancestry

To characterize the genetic ancestry of the children in the Generation R Study, all samples passing QC procedures were merged with the three genotyped panels from the HapMap Phase II release 22 build 36 including: Northwestern Europeans (CEPH collection or CEU), Sub-saharan West Africans (Yoruba or YRI) and Asians (Han Chinese from Beijing or CHB, and Japanese from Tokyo or JPT) [14, 15] using only independent autosomal SNPs (r^2^ > 0.05). In the merged dataset, pairwise identity-by-state (IBS) relations were calculated for each pair of individuals (representing the average proportion of alleles shared by those individuals) using PLINK (–genome option). In addition, principal axes of variation [or so-called genomic components equivalent to Principal Components (PCs)] were derived from this IBS matrix by multi-dimensional scaling (MDS), to characterize the variability present in the data using few variables (PLINK –cluster –mds-plot). Participants were defined as being of non- Northwestern European ancestry when deviating more than 4 standard deviations (SDs) from the CEU panel mean value in any of the first four genomic components.

#### Sample relatedness

To identify cryptic family relationships within the Generation R samples, we first removed the HapMap samples, recalculated the IBS matrix including only participants of the Generation R Study and then determined pairwise, the proportion of shared IBS alleles. By using this information and the population allele frequency, PLINK is able to estimate the number of these alleles coming from the same ancestor, known as IBD (identity-by-descent), using the methods of moments [13]. These familial relationships detected using PLINK, were validated post hoc using the recently released software REAP (Relatedness Estimation in Admixed Populations). REAP estimates IBD proportions in a similar way than PLINK. Nonetheless, it uses individual-specific allele frequencies that are calculated by conditioning on estimated individual genome-wide ancestry [16].

### Genotype imputation

A two-step genotype imputation, comprising a phasing step to resolve the haplotypes of the genotyped markers (using MACH software) and an imputation step in which unmapped SNPs are imputed to a reference panel (using Minimac software), was applied to the GWAS genotyped dataset after QC. Data was divided in marker sets across chromosomes to be processed using a parallel computing cluster. Thus far, two different reference panels have been used to impute the Generation R data: (a) HapMap Project Phase II Release 22, build 36 phasing and (b) 1000 Genomes Project (phase III release version), build 37 phasing. For the phasing of the haplotypes we used the standard parameters recommended by the MACH/minimac developers (http://genome.sph.umich.edu/wiki/Minimac) consisting of 20 iterations of the Markov sampler and 200 states (number of haplotypes that should be considered when updating each individual). Parallelization was achieved by splitting jobs on chunks across chromosomes. The window size was 2100 markers, of which 100 were flanking markers, when using assembly build 36, and a window size of 2500 markers, using 500 as flanking markers when using assembly build 37. Imputations were performed following the same chunking strategy of parallelization as mentioned for the phasing step. To evaluate genotype imputation quality we used the MACH r-squared (Rsq), metric based on the ratio of the empirically observed variance of the allele dosage to the expected binomial variance p(1 − p), assuming Hardy–Weinberg equilibrium, where p is the observed allele frequency. When imputations hold adequate information for predicting the unobserved genotypes from the observed haplotype backgrounds, this ratio should be distributed around unity [17]. By consensus an Rsq > 0.3 has been used to define sufficiently good quality for analysis [18].

#### HapMap imputations

Imputations of autosomal chromosomes to HapMap used all haplotypes available from Phase 2 of the International HapMap Project reference panel, in the so-called “cosmopolitan approach”. This combined reference panel includes 210 individuals from the CEU, YRI and CHB/JPT panels [15].

#### 1000 Genomes imputations

A second round of imputations was performed using 1000 Genomes (1KG) data—phase 3 release (http://www.sph.umich.edu/csg/abecasis/MACH/download/1000G.2012-03-14.html), which comprises the genomes of 1092 individuals from 14 populations [19]. We employed the same parameters as described for the phasing procedure in build 37, and included autosomal and chromosome X markers. Chromosome X imputations were performed separately for males and females.

### Genome wide association analysis in the Generation R Study

For illustration of possible pitfalls when using an admixed population, association analyses in the Generation R Study were performed with and without adjustment for population substructure. Additionally, we evaluated the distribution of the participant’s ethnicity along the genomic components, in order to assess the adequacy of questionnaire-based ethnicity to correct for population structure in the association models. Finally, we contrasted the two most common approaches used for correction of population stratification: (1) the traditional method of inclusion of genomic components as covariates in the association model, and (2) linear mixed models, as implemented in the publicly available software, Efficient Mixed-Model Association eXpedited (EMMAX) [20]. The genome-wide significance (GWS) threshold for the association was established at *P* < 5 × 10^−8^. For illustration, we present here examples of association results in the whole Generation R population obtained for two model phenotypes: (1) the dichotomous red hair pigmentation (a highly stratified trait) and (2) the continuous bone mineral density measured at the skull. For the former example (n = 5731), logistic association analyses ran on directly genotyped markers were corrected by four genomic components. Additionally, we used the two imputed datasets—HapMap and 1KG—to show the fine mapping resolution improvement of the genome-wide signal. For the latter skull BMD analyses (n = 4086), linear association using HapMap imputed data including twenty genomic components as covariates in the model. Further details on collection and analysis of this phenotype have been reported elsewhere [21]. For further illustration, we ran GWAS for skull BMD with equal sample sizes (n = 1909) in both the non-European and a randomly selected sample of the European subgroup of the Generation R Study, adjusting for 20 genomic components and compared results for rs13223036, reported as the top-hit in a meta-analysis of more than 9000 kids mainly from European ancestry [21].

All linear and logistic models were ran using the MACH packages (http://www.unc.edu/~yunmli/software.html) as available in the web-based tool GRIMP, which facilitates high-speed analysis of large imputed datasets making use of computational grid infrastructures [22].

## Results

### Study population

A summary of the ethnic classification based upon questionnaire of the 9749 children participating in the Generation R Study is presented on Online Resource 3. Ethnic classification was missing in 6.7 % of the population. The largest ethnic groups in the cohort were of Dutch (57 %), other European (7.4 %), Turkish (7.4 %), Surinamese (7.3 %), and Moroccan (6.4 %).

### Sample collection, biobanking and genotyping

At birth, 5908 samples were obtained from 30 ml cord blood (Generation R-1). Additionally, 320 samples were drawn during the visit to the research center at age 6 years (Generation R-2). Of the DNA samples from the collection of Generation R-1, 5815 (98.4 %) were genotyped using the Illumina HumanHap 610 Quad chip [including 620,901 markers, representing 592,532 SNPs and 28,369 copy number variation (CNV) probes]. The 1.6 % of samples not genotyped were discarded either for low quantity and/or low concentration of DNA in the stock solution as well as possible unresolved sample swaps. The extra 320 DNA samples in Generation R-2, were genotyped with the Illumina HumanHap 660 Quad chip (comprising 657,366 markers, 561,490 SNPs and 95,876 CNV probes). The genotype data were exported on forward strand for both collections. A total of 178 samples with genotyping rates lower than 97.5 % (Genome Studio sample call rate), likely arising due to low DNA quality, array problems or poor performance of agents, were excluded from the final projects (Generation R-1 and Generation R-2 sets).

### DNA quality control

#### Marker QC

CNVs reported in the manifests of the arrays, together with SNPs which could not be called in at least 95 % of the samples or with a MAF ≤ 0.001, were eliminated (Online Resources 1 and 2) before merging the Generation R-1 and Generation R-2 sets. The combined dataset, merged using only SNPs common to both platforms (n = 5809), consisted of 549,511 SNPs. No SNP was excluded in any of the two call-rate inspections. One hundred and ninety-five SNPs were removed due to differential missingness, addressing possible bias induced by batch effects between the sets. Improvements to our quality control pipeline could be implemented, as to have more stringent standards. For example, although PLINK will report alleles incompatibilities when merging datasets, these would not be detectable in case of palindromic SNPs (A/T and G/C). Therefore, since strand issues would not be detected for these type of SNPs checking allele frequencies before merging is strongly recommended. In addition, 30,971 SNPs were excluded for deviations from Hardy–Weinberg equilibrium (HWE) proportions (*P* < 1 × 10^−7^). While other causes of deviation exist, failure of this test is highly indicative of genotyping errors at a given marker [23].

#### Sample QC

Unique laboratory codes together with an anonymous person-unique study code were compared in order to identify duplicates. Fifteen duplicated samples were removed from analysis (10 from the Generation R-1 set and 5 from the Generation R-2 set). Sex inconsistencies were flagged by PLINK in 60 samples. Ten of them had incompatible sex data while the others were assessed as ambiguous. After revision of Genome Studio plots (Online Resource 4), we identified discrepancies for 15 of those samples. In total, 25 samples were excluded during this sex check. A sample genotyped call rate test, based on the remaining SNPs after merging projects, resulted in no samples exclusion. We found no individual samples with excess of heterozygosity of more than 4 SDs above the mean heterozygosity value of all samples, thus the presence of sample cross-contamination was unlikely. However, reduced heterozygosity (−4 SDs) was identified in 34 samples, possibly as result of the multiethnic background of the samples. Excess of homozygosity is typically seen in individuals from genetic isolates with large stretches of linkage disequilibrium (LD) or in populations with substructure, in which there is partial admixture as result of non-random mating, as is the case in the Generation R Study [24, 25].

### Population sub-structure

#### Genetic ancestry

**Fig. 2:**
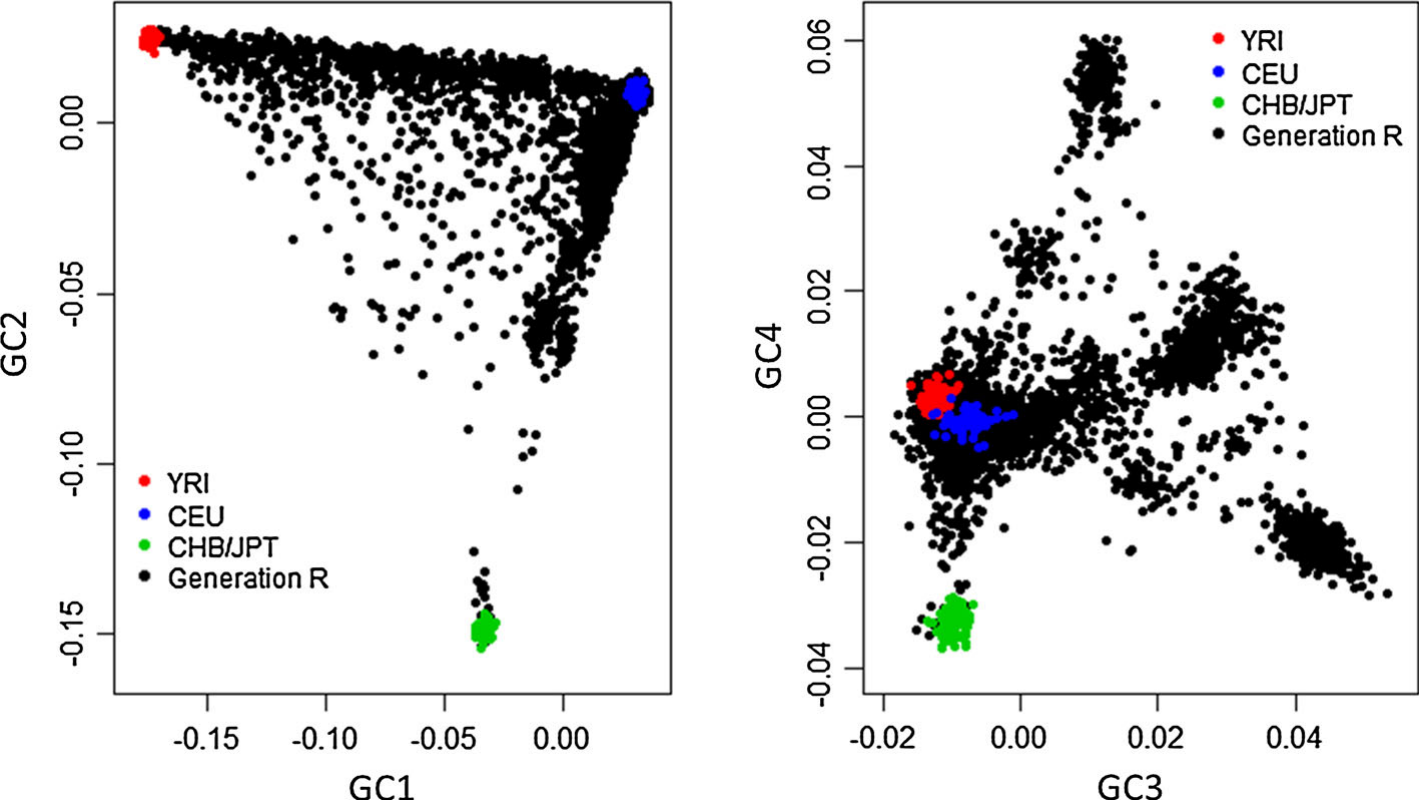
Genetic substructure of the Generation R Study. Two-dimensional plots from MDS analyses of the Generation R Study and the three initial Panels form the HapMap Project. *Left panel* First two components explaining most of the variability of the data. *Right panel* Third and fourth components explaining some of the remaining data variability

Generation R and the three HapMap panels were merged based on a common set of 36,845 independent (LD-pruned) autosomal SNPs. After calculation of pairwise IBS genetic distances between all individuals, we derived genomic components, summarizing the structure of the data into main genomic components explaining the genetic variation (Fig. 2). Approximately 50.5 % of the samples deviated more than 4 SDs from the mean CEU panel cluster on the main four components and were classified as of “Non-Northwestern European” origin. A previous release of the Generation R data from 2009, included only individuals whose samples were collected at birth, and who were classified as of Northwestern European origin (N = 2661) following the same steps mentioned above, and has been used in some publications [10, 26, 27].

#### Cryptic family relatedness

Two-hundred and eighty-nine possible pairwise sib-ships were found by IBS-sharing using PLINK (0.35 < PI_HAT < 1). Sixteen pairs of individuals shared two alleles at every locus corresponding either to monozygotic twins or a single sample processed twice. Twelve of these relations were conflicting with the registry, and thus most likely correspond to the same sample being processed twice. In these cases both samples were removed from the dataset. The four remaining pairs were twins when traced back to registries. For these true twin pairs, the sample with a lower call rate was removed from the dataset. First-degree relationships discrepant with registry (13 samples) identified using PLINK were not initially excluded. Nevertheless, they were excluded after confirmation by REAP. Visualization of kinship coefficients obtained from REAP revealed that Generation R participants are (to a large extent) unrelated. Sibling pairs are represented by the small peak around a kinship coefficient of 0.25. Yet another peak (0.025< kinship coefficient <0.0635) evidence the presence of third and fourth degree related individuals (Online Resource 5). Related individuals were not removed from the dataset to allow exclusion/inclusion in association analyses to be done specifically by phenotype availability. In addition, one more individual was recently removed for retracted informed consent. In summary, the current GWAS collection for the Generation R Study consists of samples from 5732 children.

### Genotype imputation

#### HapMap imputations

**Fig. 3:**
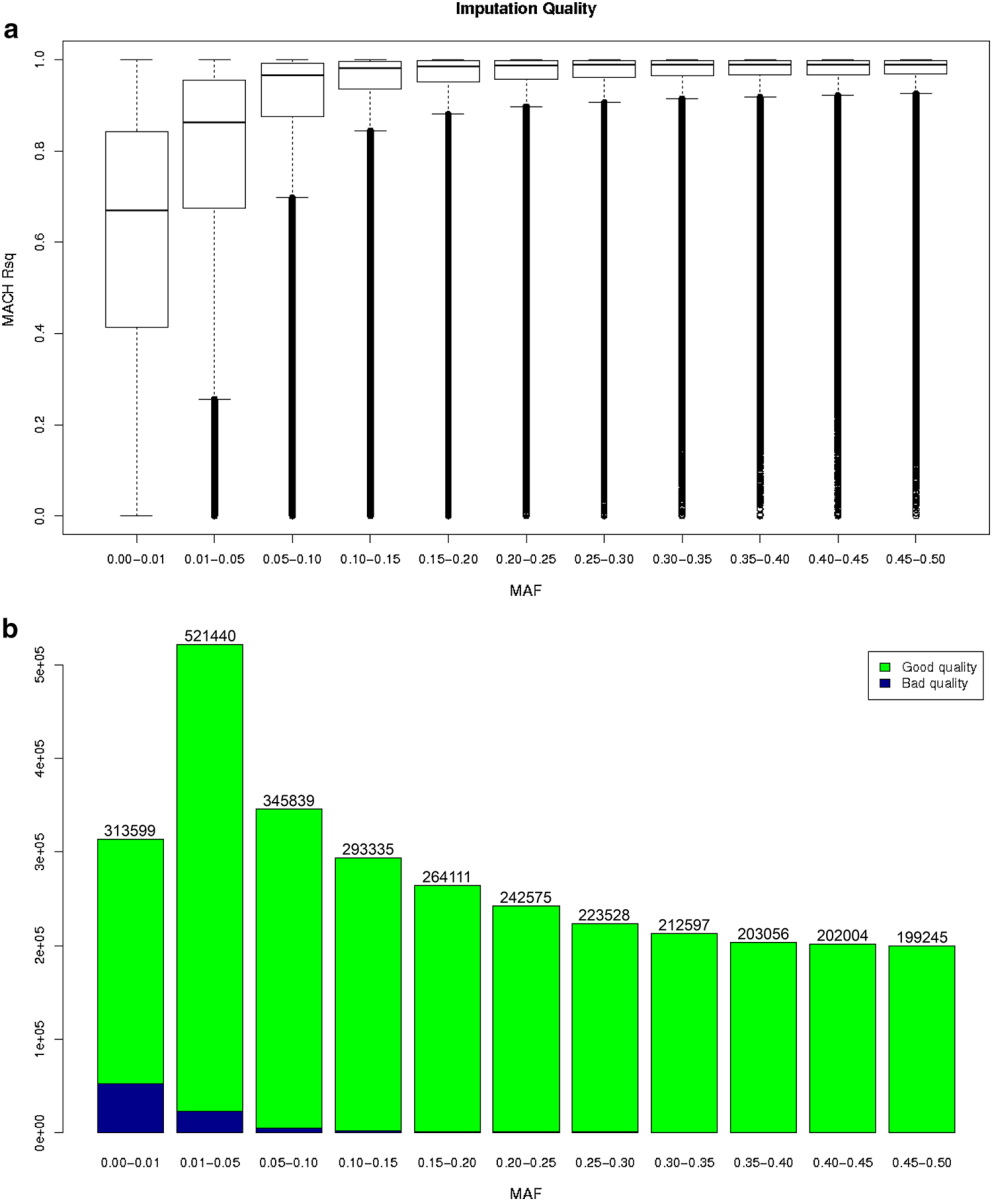
Imputation quality metrics evaluation HapMap. **a** Boxplots of the MACH Rsq in function of the MAF of the imputed SNPs. **b** Imputation quality distribution per MAF category. *Blue* and *green* denotes the poorly and well imputed SNPs based in a 0.3 quality score as threshold. 88,625 out of 3,021,329 (2.93 %) are poorly imputed SNPs (Rsq < 0.3). (Color figure online)

Using the three HapMap panels combined 3,021,329 SNPs were imputed. The MAF distribution of imputed SNPs is shown in Fig. 3. The mean Rsq for all the imputed data was 0.883, (median 0.972, IQR = 0.127); when markers with MAF < 0.01 were excluded (comprising 313,593 SNPs or 10.38 % of the markers), the mean Rsq was 0.914, (median 0.979, IQR = 0.083). Figure 3a, b shows how the increase of Rsq is proportional to the increase in the MAF of the markers. When grouping the markers into MAF bins, 83 % of the SNPs with MAF < 0.01 achieved sufficient quality, while for the other bins more than 95 % of the SNPs were well imputed. Nonetheless, there is a broad range of quality scores for SNPs in each MAF bin. Statistical dispersion is decreasing with MAF as seen by the interquartile range represented by the size of the box in each bin. Patterns of imputation quality by chromosome are shown in Online Resource 6. In general, larger chromosomes tended to be better imputed. Imputation quality was visually checked across chromosomes and the only notorious fall in Rsq was at centromeres and extremes of the telomeres, where the density of markers is low. Markers on the sex chromosomes were not imputed to the HapMap reference panel.

#### 1KG imputation

**Fig. 4:**
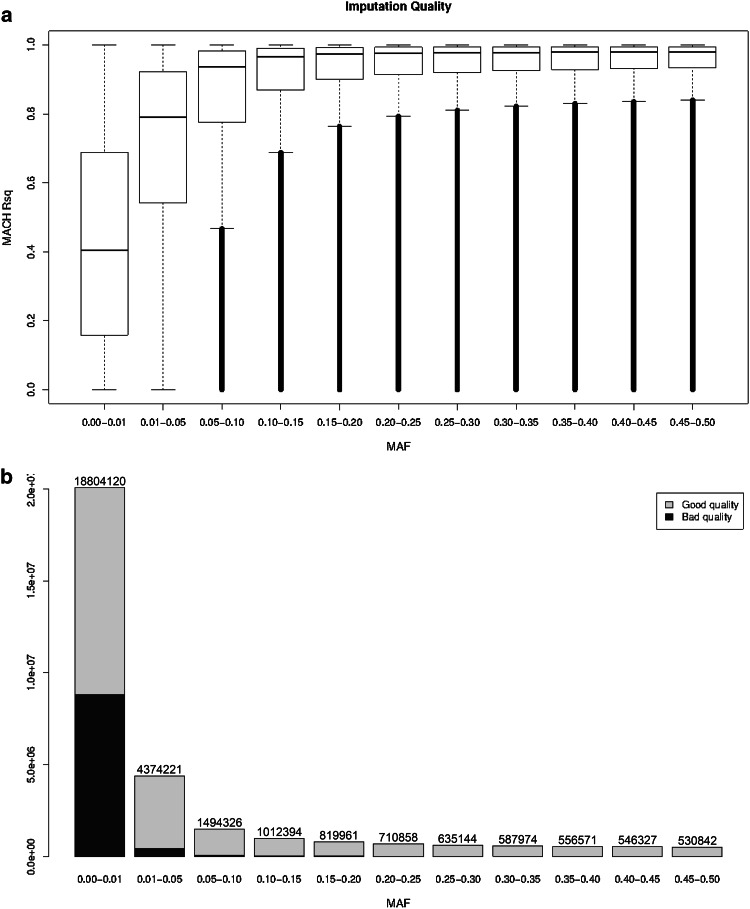
Imputation Quality metrics evaluation 1KG. **a** Boxplots of the MACH Rsq in function of the MAF of the imputed SNPs. **b** Imputation quality distribution per MAF category. *Blue* and *green* denotes the poorly and well imputed SNPs based in a 0.3 quality score as threshold. 8,263,752 out of 30,072,738 (27.4 %) are poorly imputed SNPs (Rsq < 0.3)

We were able to impute 30,072,738 autosomal variants using the 1KG reference panel, in which 28,681,763 are SNPs and 1,390,975 are insertion/deletions. The mean Rsq for all variants was 0.574 (median 0.622, IQR = 0.636); when markers with MAF < 0.01 were excluded (comprising 18,804,120 SNPs or 62.52 % of the markers), the mean Rsq increased to 0.815 (median 0.929, IQR = 0.244). Figure 4 shows an assessment of imputation accuracy by MAF. Although imputation quality was poor in the lower spectrum of allele frequencies (MAF < 0.05), 15,164,960 markers had an Rsq ≥ 0.3 and were suitable for analysis. Moreover, the number of markers comprising bins of common frequency (6,894,397 markers with MAF > 0.05) is much lower than the number of markers comprising bins of low frequency (23,178,341 markers with MAF < 0.05), which usually have low imputation quality. Online Resource 6 summarizes the performance of the imputation per chromosome. The number of SNPs imputed on chromosome X was 1,264,877, of which 903,868 (71.5 %) were rare (MAF < 0.005). As expected, quality was not as high as for the autosomal chromosomes, as a consequence of the lower number of haplotypes contributed by men in this chromosome. Considering markers of sufficient imputation quality (Rsq ≥ 0.3) on the autosomal chromosomes only, the 1KG imputation resulted in 18,874,123 more markers than those arising from the HapMap imputations including 7,892,440 markers with a MAF > 0.01. There are minimal differences in imputation quality when comparing the 2,972,940 SNPs common across the two datasets [mean Rsq, 0.886 (median = 0.972, IQR = 0.123) for the HapMap imputed dataset against 0.903 (median = 0.978, IQR = 0.097) in the 1KG imputed dataset]. When further filtering markers for MAF > 0.01 and Rsq ≥ 0.3, (resulting in 2,671,742 SNPs) the concordance rate, based on best guess genotypes, between the Hapmap and the 1KG imputed datasets was 0.983 as calculated by PLINK (using the --merge-mode 7 option).

### Correcting genome wide association analysis for ethnic background in the Generation R Study

The socio-demographic ethnic definition in the Generation R Study is based on country of birth of the parents of the participants. To evaluate the degree of potential misclassification between definitions, we assessed the agreement of the questionnaire definition with that of genetic ancestry, using genomic components (Online Resource 7). Groups classified as being of European and Dutch origin have historically undergone high waves of migration during the 1960s, 1970s or early 1980s. As a consequence, a scattered distribution across the genomic components axes was observed instead of a uniform one. A similar pattern was also observed for participants of Surinamese origin, arising from two clearly differentiated ethnic groups, the Hindustani and the Creoles.

**Fig. 5:**
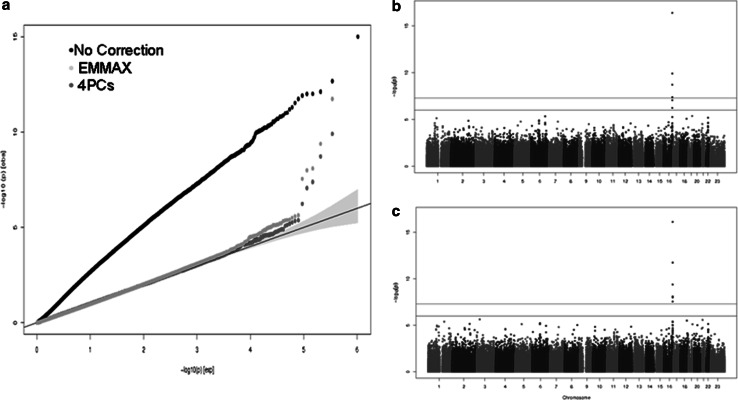
Genome-wide association of *red-hair* pigmentation in the Generation R cohort. **a** Q–Q plot showing the inflation of the test statistics when correction for data structure is not applied (*black dots*) and the slightly lower power when genomic components correction is applied (*red dots*) in comparison with the EMMAX model (*green dots*). **b** Manhattan plots of the *red-hair* pigmentation GWAS in the Generation R Study using adjustment for genomic components. **c** Manhattan plots of the *red-hair* pigmentation GWAS in the Generation R Study using a linear mixed model as implemented in EMMAX. (Color figure online)

Statistical approaches based on EMMAX and genomic components were tested for two different traits.). There is no evidence of major degrees of residual population stratification in the GWAS results for red hair color (Fig. 5 and Online resource 8), within the Generation R Study (196 children with red hair (3.4 %) as gauged in the QQ-plots (no early deviation from the test statistic or *p* value distribution) and genomic inflation factors (GIF) close to unity for both EMMAX (GIF = 0.994) and genomic components correction (GIF = 0.999). In contrast, when no adjustment for population stratification was employed, very early (artefactual) deviation was seen in the QQ plot, erroneously indicating that the vast majority of markers across the genome were associated with red hair pigmentation (Fig. 5). After correction for population stratification, only the markers on chromosome 16q24.3 mapping in the vicinity of *MCR1* reached GWS, variants in this gene largely explain the presence of red hair pigmentation [28]. GWAS based on the imputed data gave rise to similar results, but showed an even higher number of SNPs underlying the *MCR1* associated signal. Furthermore, the leading SNP on these analyses was a missense variant rs1805007, *P* < 1 × 10^−20^, reported previously as associated with this trait [29], which was not present in the genotyped data (Online Resource 9). QQ-plots from the skull BMD GWAS show adequate correction for population stratification (Online Resource 10). Power for both EMMAX and genomic components is similar in the two tested traits, as gauged by the number of GWS signals and their significant level (Online resources 8 and 11). Moreover the effect size of skull BMD associated SNPs is practically identical across the two approaches.

Skull BMD analysis for equal sets of European and Non-European children shows a GWS signal in the *WNT16/CPED1* locus only in the Non-European children, although similar direction for the leading SNPs was found in both sets (Online Resource 12). We compared the association results for rs13223036 in this locus. The frequency of the effect allele in Europeans was 0.622, while it was 0.695 in non-Europeans. The effect size differed by ~27 % of the effect size in the European group (β = 0.15, *P* = 6.8 × 10^−6^), being stronger and more significant in the non-European set (β = 0.19, *P* = 2.5 × 10^−8^).

## Discussion

In summary, we have described the methodology used to genotype, impute, and analyze data for association with phenotypes in the multiethnic Generation R Study, addressing a number of practical issues that arise in implementing imputation-based association for a multiethnic cohort.

Our genome-wide genotyped data, ready for analysis after quality control (QC), comprises information for 518,245 markers in 5732 individuals of different ethnic backgrounds, which is available in the most common genome builds (i.e. 36 and 37). Enrichment by imputation of our genotypes, following a cosmopolitan approach, resulted in an increment of the number of markers of about 5.7 times for the HapMap imputed data and 46 times for the 1KG imputed data (Rsq > 0.3).

The Generation R Study withholds a special setting determined by the admixed nature of its population confined within a restricted area. This encompasses analytical challenges as well as opportunities to design genetic studies, which take advantage of such characteristics. A joint analysis including all Generation R participants represents a considerable increase in power of the design, as about half of the study population is of non-Northwestern European background. While, increment in sample size will in principle boost the power of the study, differences in allele frequency or LD relationships between the variants merit further interpretation, as shown in the example of skull BMD with equal number of individuals for both European and non-European sets. Decrease in power due to the use of an admixed population can appear when the association is confined to one of the subpopulations (especially if small) either because of differential tagging or due to the effect of secondary signals [30].

GWAS meta-analyses are expanding to include Non-European populations (i.e. Latinos, African-Americans, etc.) with adequate methodology lagging behind due to scarce available software for the processing and analysis of multiethnic data. For example, as the most used software, PLINK [13], relies on the assumption of homogeneous populations it cannot be applied directly to establish family relatedness in multiethnic cohorts. PLINK routine results in an overestimation of relatedness between ancestrally similar individuals. Alternatively, REAP [16] employs a routine that considers the presence of more than one ancestral population and accounts for it in the calculation of IBD probabilities. Nevertheless, in our range of interest for QC purposes—greater than second degree relatives—we found no misclassification of the degree of relationship in the samples. Yet, in situations where high sensitivity is required (e.g. for the assessment of distant relatedness and/or fine pedigree structure), REAP is recommended in studies with admixed populations.

Choosing the optimal panel to impute the GWAS data of a multiethnic population is critical. For the Generation R Study, we have employed the so-called “cosmopolitan approach”, which has become the preferred approach after the release of the 1000 Genomes Project panel [19]. Notably, nowadays all studies are being imputed to the whole 1KG reference panel regardless of the background of the population. Introducing such a combination of reference panels, which achieve very large sample sizes of sequencing reference sets, has been shown to improve imputation accuracy [31–33]. This is mainly beneficial for the imputation of rare variants, which have probably arisen recently and are highly population specific.

New denser reference panels for imputation are becoming available achieving a better characterization of human genetic variation [7, 19, 34]. The 1KG project data significantly increased the genomic coverage providing more variants suitable to be analyzed in a new phase of the GWAS era [19], with already few reports of novel findings [6, 35, 36], yet to be embraced at a larger scale. Despite the higher density of markers in the 1KG, only ~63 % of the markers achieved good quality as compared to 97 % of the HapMap imputed markers. Nonetheless, the low imputation performance observed in 1KG markers is a consequence of the large amount of low-frequency and rare markers in the panel in low LD with the tagging SNPs in the array, which are thus, difficult to impute. When the analysis is limited to common variants (MAF > 0.05) present in both datasets (n = 2,144,906) the imputation quality was somewhat higher in the 1KG (mean Rsq = 0.954, median = 0.989, IQR = 0.037) than the HapMap panel (mean Rsq = 0.947, median = 0.986, IQR = 0.046), an slight improvement reflecting better imputation arising from a more dense set of markers and a larger reference panel. Special methods for imputation of admixed populations such as MACH-Admix, have also emerged [37], claiming better performance in admixed population and should be part of future studies.

Ethnic background, as assessed by questionnaire did not match the distribution of the samples in the genomic components, mainly because it does not allow for the identification of the third generation participants, i.e. the grand children of those who originally migrated to the country, and thus groups together children that are genetically divergent as shown in Online Resource 7. This comparison together with analysis of different traits, indicate that the genetic structure of studies, such as the Generation R Study, cannot be accounted for by considering the ethnic group definitions based on questionnaire data alone. Another practical advantage when using genomic components to adjust GWAS, is the possibility to include participants even when no information of the parents’ country of origin is available. Although in other studies this percentage might be larger, in the population under study, <7 % of the information on ethnicity from the questionnaire was missing. The ethnic distribution of the remaining children is in agreement with the ethnic demography of the city of Rotterdam [11] and thus we found no evidence of a systematic non-response.

We chose red hair pigmentation as an example of a highly stratified trait since it is more common in countries in the north of Europe and selected against in Africa due to higher sensitivity of its carriers to UV rays. As shown in Fig. 5, if adjustment for population stratification is not used, alleles with different frequencies in Africa and the North of Europe would spuriously show an association with the trait. Instead our association results show that both genomic components and linear mixed models strategies cope well with the substructure of the data and yield similar results. This conclusion can also be derived from the skull BMD GWAS, where even magnitude of effect sizes can be reliably compared. Thus, across all tested scenarios subtle differences emerged not justifying the use of the more computational intensive EMMAX approach. Tests done on other traits such as height, fractional exhaled nitric oxide, site-specific BMD (total body, skull, arms and legs) produced similar results using EMMAX as those published before using genomic components [21, 38, 39].

It is important to emphasize possible drawbacks of both association strategies. Those of using EMMAX include: (1) for discrete traits obtained betas cannot be translated to odd ratios, given the statistical model applied and (2) its requirement for PLINK files, prevents the use of allelic dosages for analysis. On the other hand, since genomic components are calculated based on the variability of the input data, it is important to generate specific sets of components for particular subsets of the data when working with structured populations. Moreover, while using between two and four genomic components is common practice, the number of genomic components needed to control for population stratification is trait-specific (i.e. dependent on the actual genetic architecture of the trait) [40]. The GIF is an indicator of the degree of inflation of the test statistic due to true signals, cryptic relatedness, assay bias and/or population stratification. Hence, assessment of the GIF is instrumental to determine the needed number of genomic components to be used as covariates in the models. This strategy is not confined to admixed populations and should be assessed even in homogenous populations. In the examples mentioned above, both height and BMD needed up to ten genomic components (data not shown) to reach an acceptable GIF < 1.1 [18].

Although the general problem of stratification, differential ethnic allele frequencies, has been successfully addressed in our cohort by the use of genomic components or linear mixed models, the ethnic differences in patterns of correlation between the underlying casual variant and the surrounding SNPs which are under study (LD), can still induce to false-negative findings [41].

As single-center GWAS are usually underpowered, the standard strategy in the field is meta-analysis, the combination of results from multiple independent studies, increasing sample size and reducing false-positive findings. Frequently, pooling studies from ethnically diverse populations within a single transethnic meta-analysis can be challenging. To cope with this, specialized software such as MANTRA, which allows effect size to vary across different populations, has been developed [42]. The same strategy could also be applied to multiethnic studies such as Generation R, if clear boundaries between different ethnic groups forming part of the study population could be established. However, this is not plausible in our highly admixed population.

The complex structure of the Generation R Study, where admixture of individuals cannot be easily discerned just by assessing the combination of two ancestral populations, constrains the application of admixture mapping, which is an important limitation of our study. Further, in the current setting of the Generation R Study, the small sample size resulting from defining well characterized ethnic groups (of non-European background) is insufficient to allow fine mapping of variants underlying complex traits, typically withholding weak genetic effects. Yet, with new approaches being developed [39], this analytical methodology should be further implemented.

The Generation R Study is unusual in the international arena due to its size, age range, quality of data and longitudinal study design, but particularly due to its multiethnic nature. These characteristics represent the main strengths of the cohort, allowing among others, the generalizability of findings and ethnic comparisons in epidemiological research, although complex routines might be required for genetic association analysis.

In summary, we have described the methods used for generating the GWAS data of the Generation R Study, as well as general strategies for imputation and analysis within a multiethnic setting. Such strategies have allowed the Generation R Study to take part in several consortia and collaborations, which have successfully identified genetic factors underlying an ample range of complex traits.

## Electronic supplementary material

Below is the link to the electronic supplementary material.

Supplementary material 1 (PDF 90 kb)

Supplementary material 2 (PDF 127 kb)

Supplementary material 3 (PDF 79 kb)

Supplementary material 4 (PDF 101 kb)

Supplementary material 5 (PDF 94 kb)

Supplementary material 6 (PDF 65 kb)

Supplementary material 7 (PDF 83 kb)

Supplementary material 8 (PDF 35 kb)

Supplementary material 9 (PDF 54 kb)

Supplementary material 10 (PDF 51 kb)

Supplementary material 11 (PDF 76 kb)

Supplementary material 12 (TIFF 171 kb)
