# Supplementary material for: Challenges in conducting genome-wide association studies in highly admixed multi-ethnic populations: the Generation R Study
Source: Eur J Epidemiol. 2015 Mar 12;30(4):317–30. doi: 10.1007/s10654-015-9998-4 (PMC4385148; doi:10.1007/s10654-015-9998-4)
Supplement: Supplementary file 1 — Supplementary material 1 (PDF 90 kb) [file 10654_2015_9998_MOESM1_ESM.pdf]

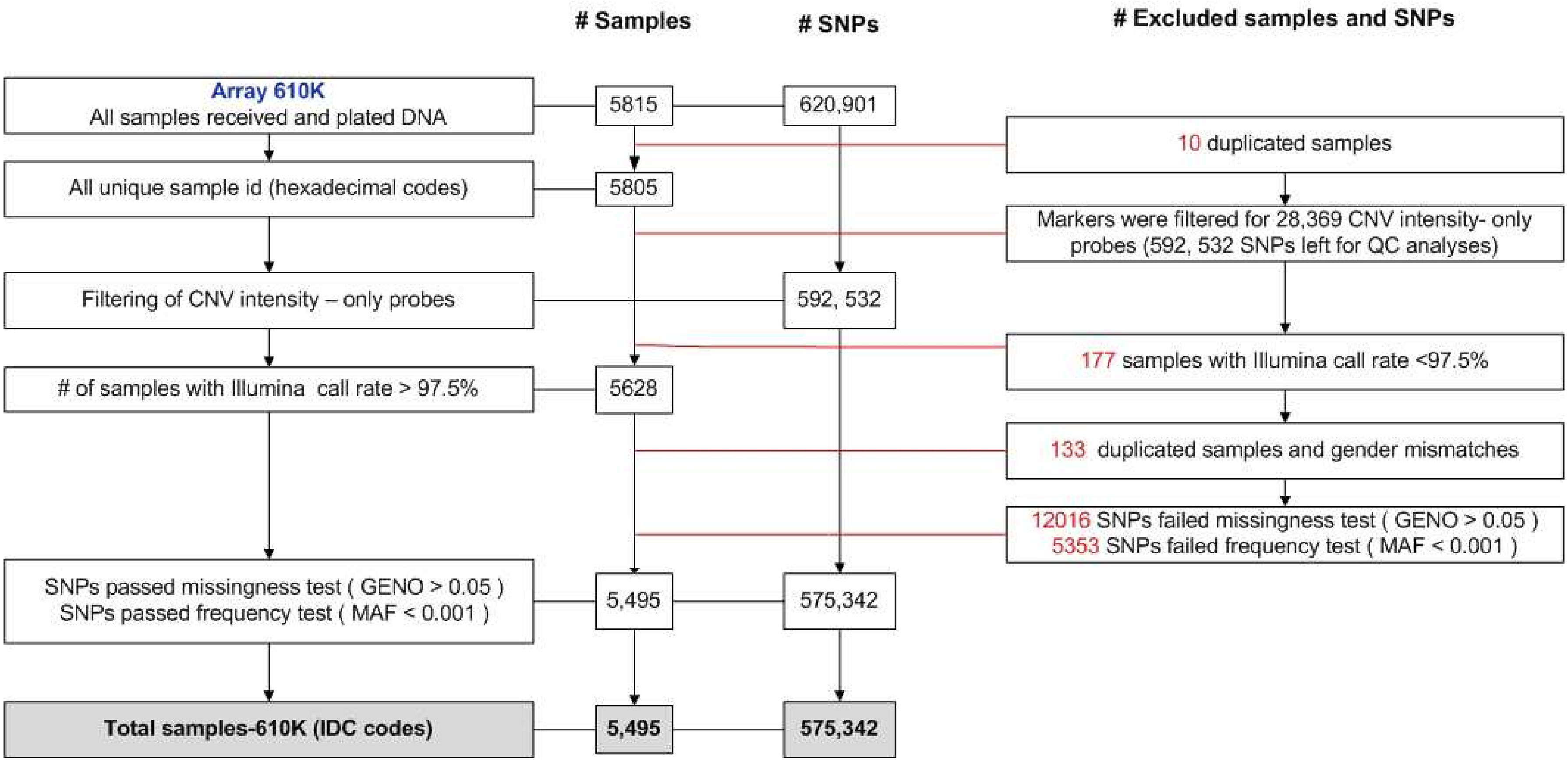


**Online Resource 1. Flowchart overview of the Generation R-1 set.** Quality control of samples collected at birth from cord blood (Generation R-1) and processed in platform Illumina HapMap 610-K before merging the projects. Red font denotes exclusion of either SNPs or samples from the dataset in the different QC steps.
