# Supplementary material for: Challenges in conducting genome-wide association studies in highly admixed multi-ethnic populations: the Generation R Study
Source: Eur J Epidemiol. 2015 Mar 12;30(4):317–30. doi: 10.1007/s10654-015-9998-4 (PMC4385148; doi:10.1007/s10654-015-9998-4)
Supplement: Supplementary file 2 — Supplementary material 2 (PDF 127 kb) [file 10654_2015_9998_MOESM2_ESM.pdf]

a.

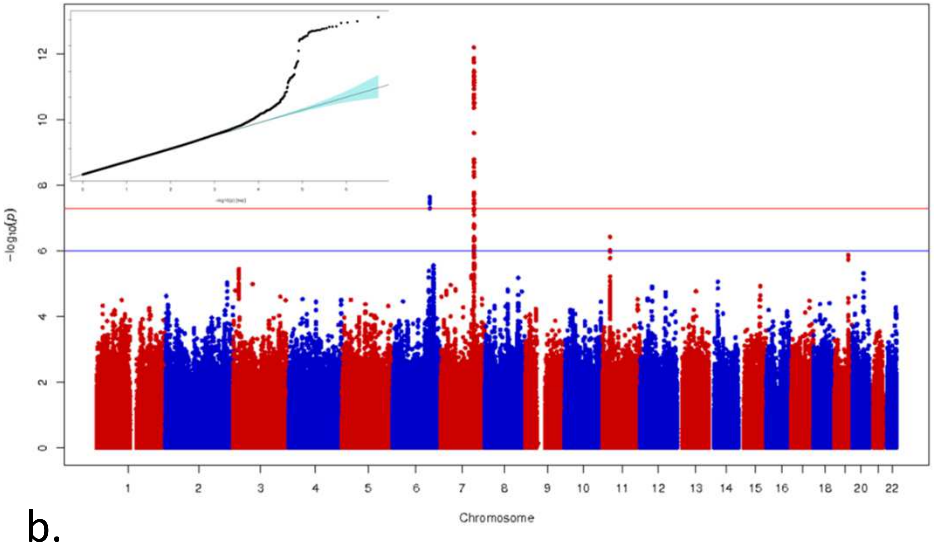

b.

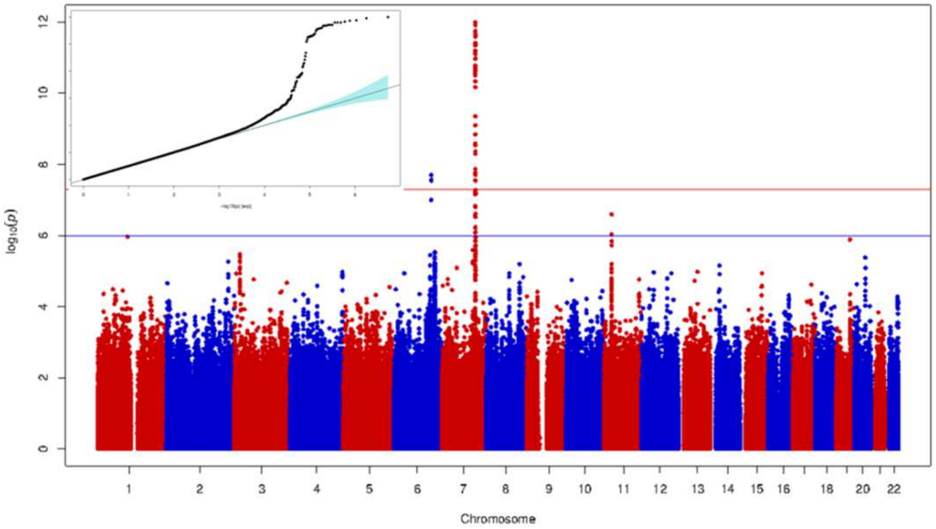

Online Resource 10. GWAS of skull BMD in the Generation R cohort Q-Q plot by MAF and Manhattan Plot for the association analysis based on adjustment for 20 genomic components. **b**. Q-Q plot by MAF and Manhattan Plot for the association analysis based on Linear Mixed Models (EMMAX).
