# Supplementary material for: Challenges in conducting genome-wide association studies in highly admixed multi-ethnic populations: the Generation R Study
Source: Eur J Epidemiol. 2015 Mar 12;30(4):317–30. doi: 10.1007/s10654-015-9998-4 (PMC4385148; doi:10.1007/s10654-015-9998-4)
Supplement: Supplementary file 3 — Supplementary material 3 (PDF 79 kb) [file 10654_2015_9998_MOESM3_ESM.pdf]

Online Resource 11. Skull BMD association results comparison. Analysis summary for the top hit SNPs associated with skull BMD in the Generation R Study, both by mixed models (EMMAX) and adjustment by genomic components.

SNP	CHR	BP	EA	OA	EAF	BETA_20PCs	P_20PCs	BETA_EMMAX	P_EMMAX	n_total
rs13223036	7	1.21E+08	T	G	0.655	0.167	6.21E-13	0.164	1.02E-12	4086
rs1917113	7	1.21E+08	G	A	0.661	0.164	1.29E-12	0.163	1.23E-12	4086
rs6954757	7	1.21E+08	G	A	0.661	0.163	1.59E-12	0.162	1.77E-12	4086
rs6466769	7	1.21E+08	A	G	0.661	0.163	1.76E-12	0.162	1.86E-12	4086
rs10500083	7	1.21E+08	T	C	0.649	0.164	3.25E-12	0.163	2.23E-12	4086
rs2272196	7	1.21E+08	G	A	0.649	0.164	3.41E-12	0.163	2.56E-12	4086
rs7795692	7	1.21E+08	A	G	0.649	0.164	3.54E-12	0.163	2.56E-12	4086
rs10480747	7	1.21E+08	A	C	0.659	0.163	4.45E-12	0.163	2.57E-12	4086
rs11771945	7	1.21E+08	C	G	0.656	0.162	6.15E-12	0.162	3.81E-12	4086
rs11765163	7	1.21E+08	A	T	0.656	0.162	6.20E-12	0.162	3.81E-12	4086
