# Supplementary material for: Challenges in conducting genome-wide association studies in highly admixed multi-ethnic populations: the Generation R Study
Source: Eur J Epidemiol. 2015 Mar 12;30(4):317–30. doi: 10.1007/s10654-015-9998-4 (PMC4385148; doi:10.1007/s10654-015-9998-4)
Supplement: Supplementary file 4 — Supplementary material 4 (PDF 101 kb) [file 10654_2015_9998_MOESM4_ESM.pdf]

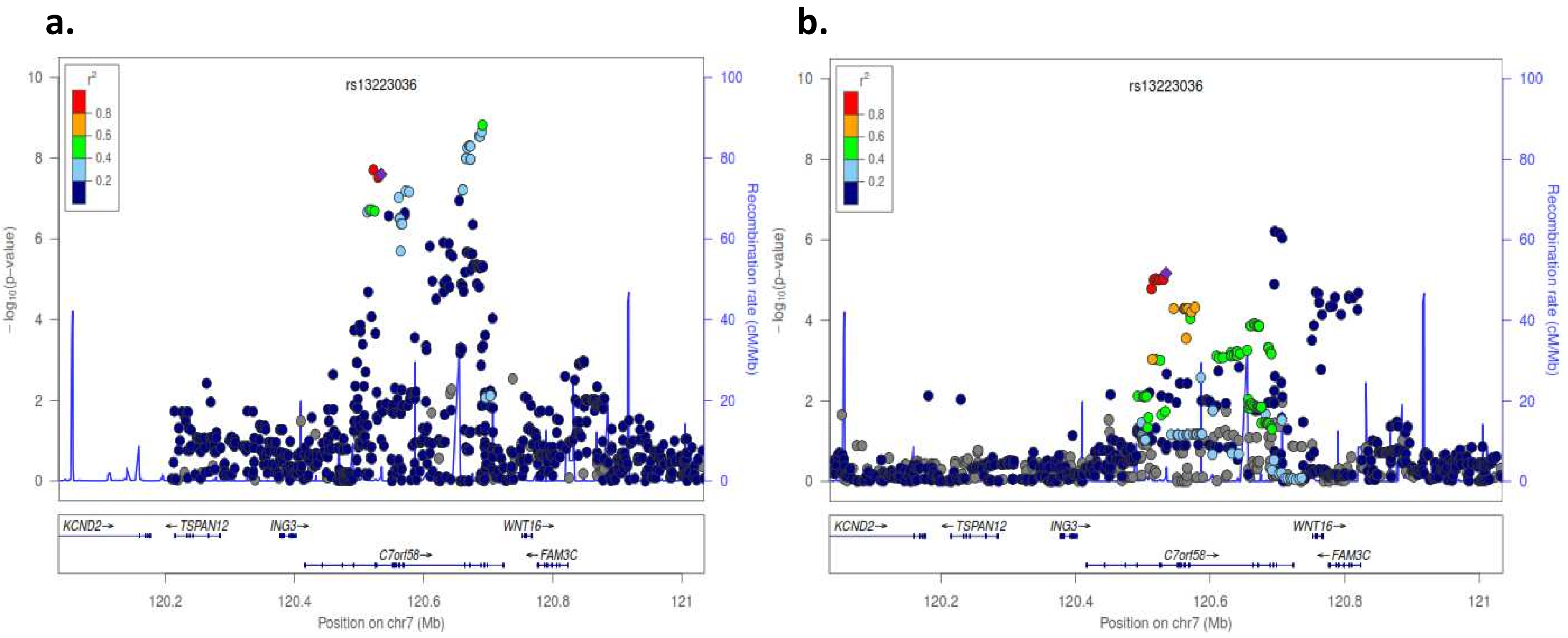

Online Resource 12. Regional Association Plot for skull BMD in the *WNT16/CPED1* loci. GWAS using genomic components approach. SNP rs13223036 is denoted by a diamond. Different colors indicate varying degrees of pair-wise linkage disequilibrium between the top SNP and all other genotyped SNPs. **a.** Non-European children (n=1,909). Genetic coordinates are per Hap-Map ref. 22 b.36-YRI. **b.** European children (n=1,909). Genetic coordinates are per Hap-Map ref. 22 b.36-CEU.
