# Supplementary material for: Challenges in conducting genome-wide association studies in highly admixed multi-ethnic populations: the Generation R Study
Source: Eur J Epidemiol. 2015 Mar 12;30(4):317–30. doi: 10.1007/s10654-015-9998-4 (PMC4385148; doi:10.1007/s10654-015-9998-4)
Supplement: Supplementary file 5 — Supplementary material 5 (PDF 94 kb) [file 10654_2015_9998_MOESM5_ESM.pdf]

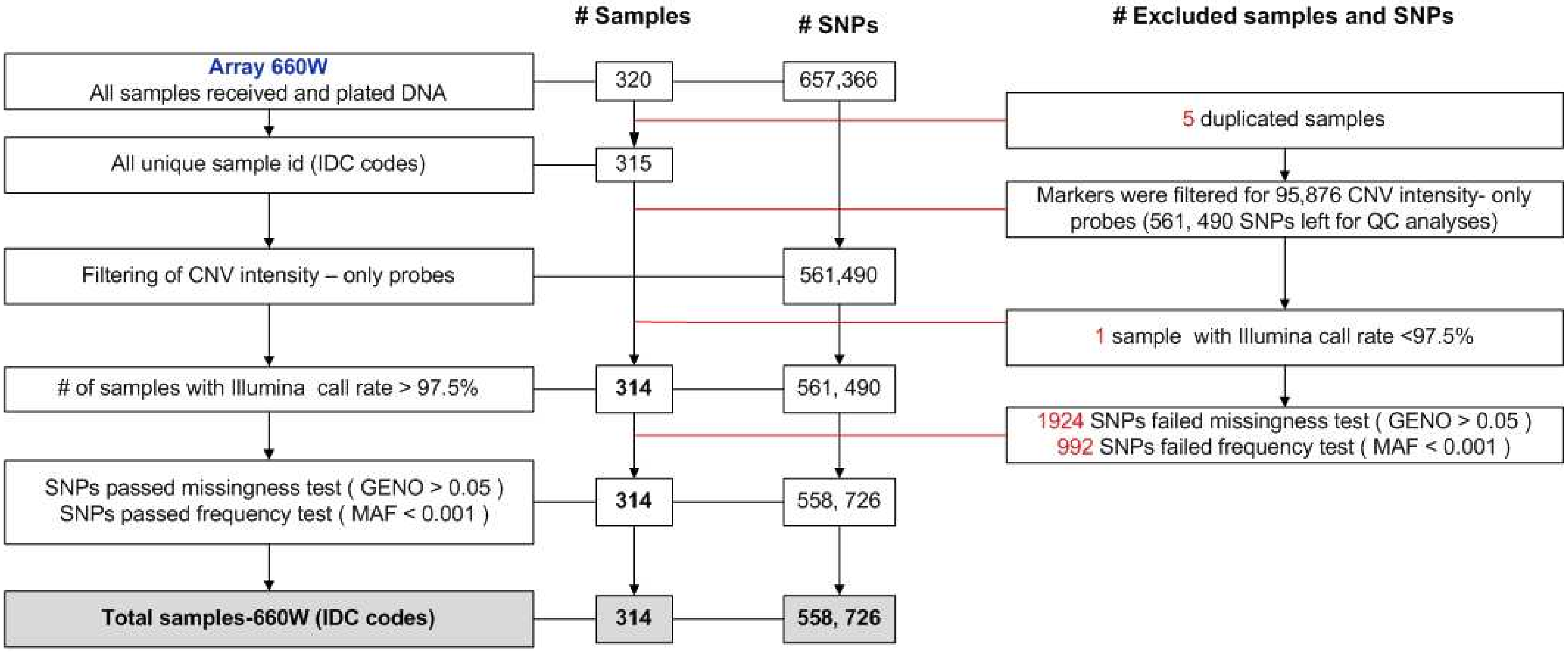

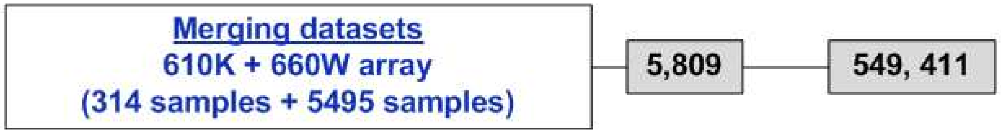

Online Resource 2. Flowchart overview of the Generation R-2 set and merging of the two datasets. Quality control of samples collected at 6 years from peripheral blood (Generation R-2) and processed in platform Illumina HapMap 660-W and merging of the projects. Red font denotes exclusion of either SNPs or samples from the dataset in the different QC steps.
