# Supplementary material for: Challenges in conducting genome-wide association studies in highly admixed multi-ethnic populations: the Generation R Study
Source: Eur J Epidemiol. 2015 Mar 12;30(4):317–30. doi: 10.1007/s10654-015-9998-4 (PMC4385148; doi:10.1007/s10654-015-9998-4)
Supplement: Supplementary file 6 — Supplementary material 6 (PDF 65 kb) [file 10654_2015_9998_MOESM6_ESM.pdf]

Ethnicity as defined by the Generation R Study. Ethnicity of the children part of the Generation R birth cohort.

Ethnicity	Frequency	Percent	Cumulative Percent
Dutch	4897	50.2	**53.8**
Indonesian	41	0.4	**54.3**
Cape Verdian	252	2.6	**57.1**
Moroccan	626	6.4	**64**
Dutch Antilles	358	3.7	**67.9**
Surinamese	707	7.3	**75.7**
Turkish	722	7.4	**83.6**
African	249	2.6	**86.3**
American western	61	0.6	**87**
America non western	157	1.6	**88.7**
Asia western	9	0.1	**88.8**
Asia non western	280	2.9	**91.9**
European	719	7.4	**99.8**
Oceania	16	0.2	**100**
Total	9094	93.3	
Missing	655	6.7	
	9749	100	

American western: United States of America and Canada
American Non-Western: Other countries in the American Continent except United States of America and Canada
Asian Western: Japan
Asian Non-Western: Other countries in the Asian continent except for Japan and Indonesia
