# Supplementary material for: Challenges in conducting genome-wide association studies in highly admixed multi-ethnic populations: the Generation R Study
Source: Eur J Epidemiol. 2015 Mar 12;30(4):317–30. doi: 10.1007/s10654-015-9998-4 (PMC4385148; doi:10.1007/s10654-015-9998-4)
Supplement: Supplementary file 7 — Supplementary material 7 (PDF 83 kb) [file 10654_2015_9998_MOESM7_ESM.pdf]

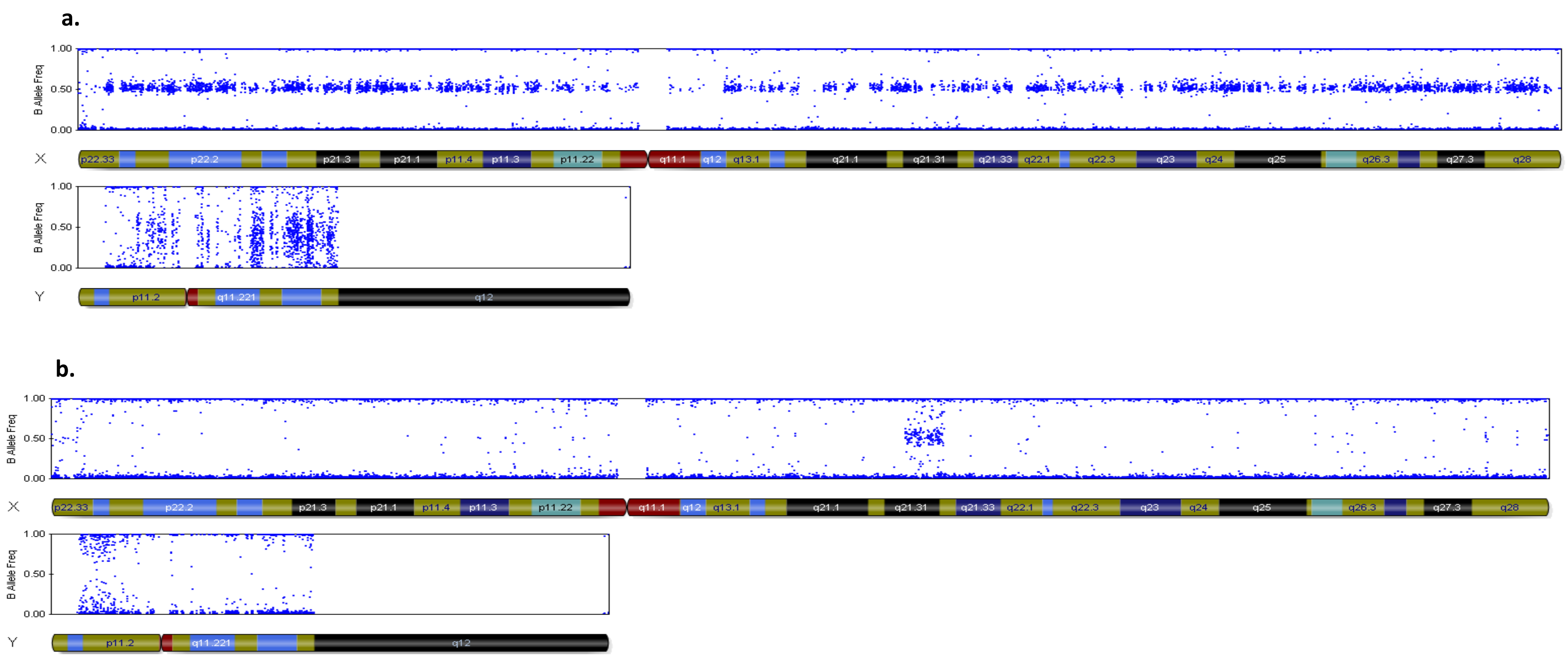

Heterozygosity checks on chromosomes X and Y. The heterozygosity output from Genome studio allows the identification of both males and females. **a.** A normal female: homozygous and heterozygous X SNPs. The disperse patter for Y-chromosome markers are seen in all females. **b**. A normal male: homozygous X SNPs and homozygous Y SNPs (few heterozygous SNPs).
