# Supplementary material for: Challenges in conducting genome-wide association studies in highly admixed multi-ethnic populations: the Generation R Study
Source: Eur J Epidemiol. 2015 Mar 12;30(4):317–30. doi: 10.1007/s10654-015-9998-4 (PMC4385148; doi:10.1007/s10654-015-9998-4)
Supplement: Supplementary file 8 — Supplementary material 8 (PDF 35 kb) [file 10654_2015_9998_MOESM8_ESM.pdf]

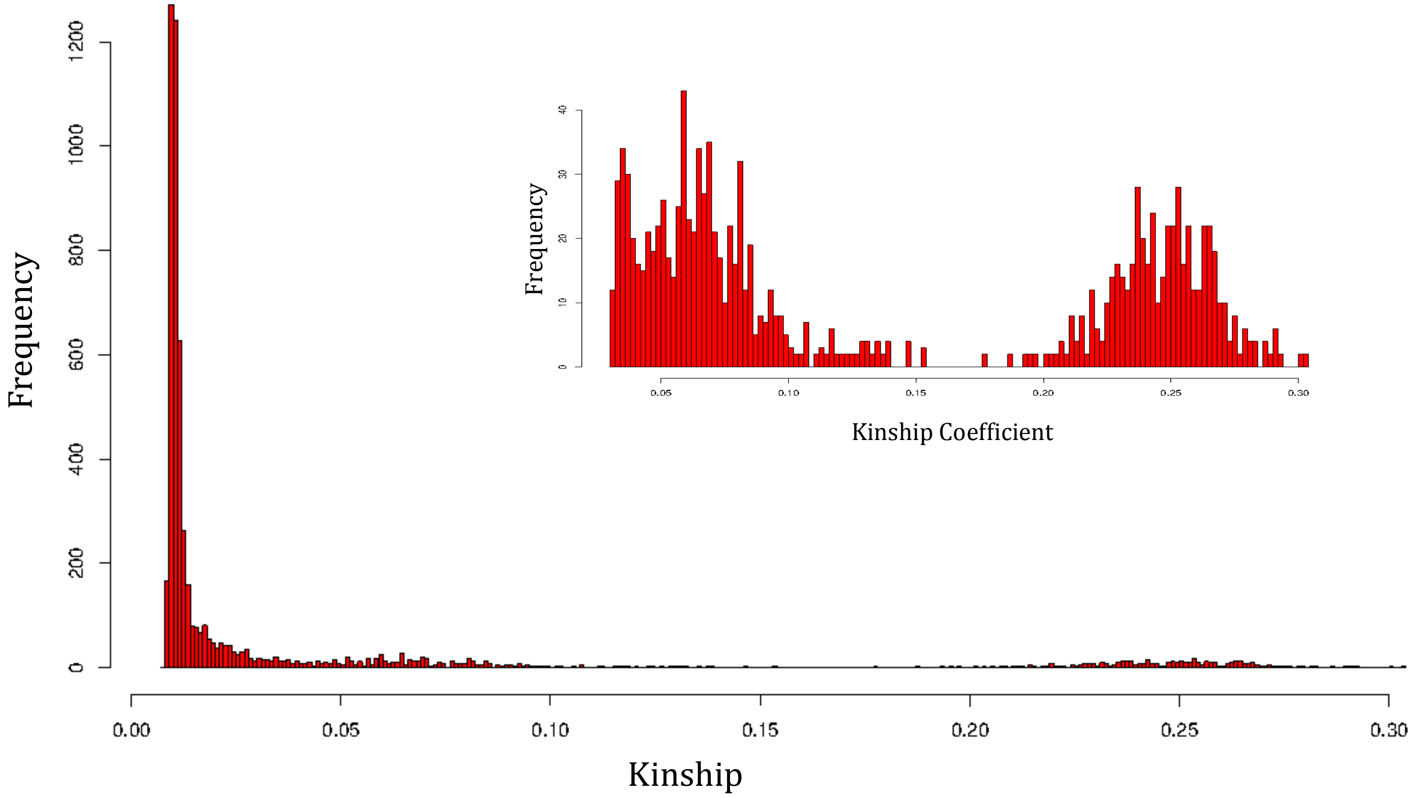

Histogram showing the distribution of pairwise kinship coefficients (smaller picture is zoomed into the part of the graph in which the coefficient is greater than 0.03125, n=1593). The peak over 0.25 represents siblings. The peak close to 0.25 second degree relatives and at 0.0625 third degree relatives.
