# Supplementary material for: Challenges in conducting genome-wide association studies in highly admixed multi-ethnic populations: the Generation R Study
Source: Eur J Epidemiol. 2015 Mar 12;30(4):317–30. doi: 10.1007/s10654-015-9998-4 (PMC4385148; doi:10.1007/s10654-015-9998-4)
Supplement: Supplementary file 9 — Supplementary material 9 (PDF 54 kb) [file 10654_2015_9998_MOESM9_ESM.pdf]

MACH Rsq patterns by chromosome. The recommended threshold for Good–quality SNPs to be used for downstream analysis is Rsq>=03.

	CHROMOSOME																						
**MACH R2**	**1**	**2**	**3**	**4**	**5**	**6**	**7**	**8**	**9**	**10**	**11**	**12**	**13**	**14**	**15**	**16**	**17**	**18**	**19**	**20**	**21**	**22**	**TOTAL**
HapMap																							
**Bad quality**	8200	6328	4447	5069	4890	4986	4833	3579	4258	4613	4096	5025	3332	2843	3366	3853	2961	3025	2665	2887	1423	1946	**88625**
**Good quality**	228667	261026	197504	186055	192896	209995	162037	169281	139218	162141	151521	141297	121948	96742	82861	82493	66898	90374	40657	73289	37845	37959	**2932704**
**%R2<0.3**	3.46%	2.37%	2.20%	2.65%	2.47%	2.32%	2.90%	2.07%	2.97%	2.77%	2.63%	3.43%	2.66%	2.86%	3.90%	4.46%	4.24%	3.24%	6.15%	3.79%	3.62%	4.88%	**2.93%**
1KG																							
**Bad quality**	657829	685548	558906	597384	504643	505472	505524	436387	339923	370793	402498	386277	268817	263920	264367	305205	292029	233707	259447	188387	116460	122388	**8265911**
**Good quality**	1697611	1898088	1609139	1571480	1485993	1420742	1247820	1279072	958421	1115971	1083127	1053925	817268	725442	619541	637635	526134	621840	392776	479743	293173	271886	**21806827**
**%R2<0.3**	**27.93%**	**26.53%**	**25.78%**	**27.54%**	**25.35%**	**26.24%**	**28.83%**	**25.44%**	**26.18%**	**24.94%**	**27.09%**	**26.82%**	**24.75%**	**26.68%**	**29.91%**	**32.37%**	**35.69%**	**27.32%**	**39.78%**	**28.20%**	**28.43%**	**31.04%**	**27.49%**
