# Supplementary material for: Challenges in conducting genome-wide association studies in highly admixed multi-ethnic populations: the Generation R Study
Source: Eur J Epidemiol. 2015 Mar 12;30(4):317–30. doi: 10.1007/s10654-015-9998-4 (PMC4385148; doi:10.1007/s10654-015-9998-4)
Supplement: Supplementary file 10 — Supplementary material 10 (PDF 51 kb) [file 10654_2015_9998_MOESM10_ESM.pdf]

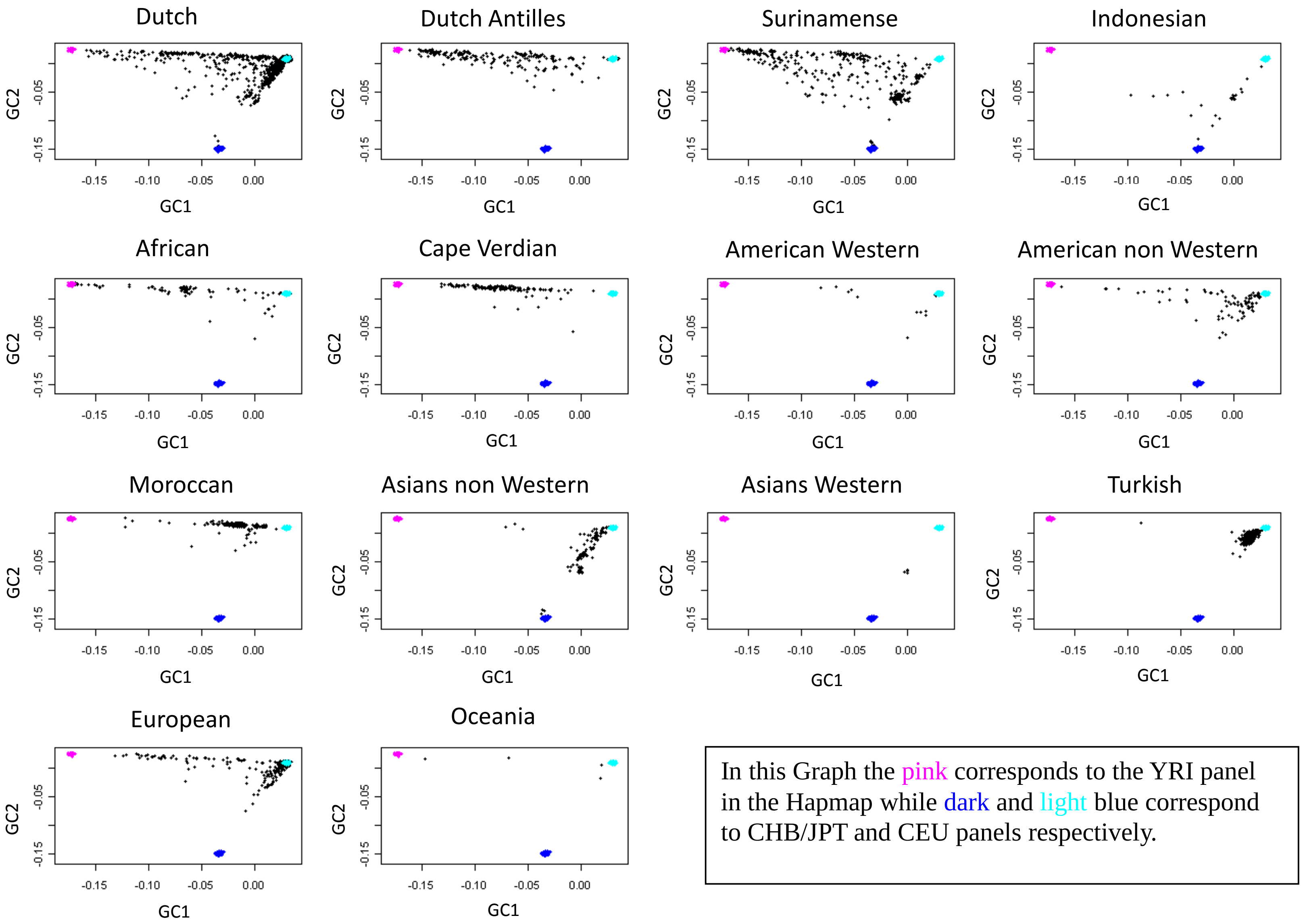

Online Resource 7. Ethnic sub-structure of the Generation R Study. Two-dimensional plots of the first and second dimensions from a MDS performed with an Identical By State (IBS) distance matrix computed between pairs of individuals, denoted as GC. Each panel represents a different ethnicity as calculated by the classification of Statistics Netherlands
