# Supplementary material for: Challenges in conducting genome-wide association studies in highly admixed multi-ethnic populations: the Generation R Study
Source: Eur J Epidemiol. 2015 Mar 12;30(4):317–30. doi: 10.1007/s10654-015-9998-4 (PMC4385148; doi:10.1007/s10654-015-9998-4)
Supplement: Supplementary file 11 — Supplementary material 11 (PDF 76 kb) [file 10654_2015_9998_MOESM11_ESM.pdf]

Online Resource 8. Red hair association results comparison. Analysis GIF and p-values for the top hit SNPs associated with red hair color in the Generation R Study, both by mixed models (EMMAX) and adjustment by genomic components.

	**EMMAX**	**4 PCs**
GIF	0.994	0.999
Top-Hits P-value		
rs258322	7.53E-17	4.03E-17
rs8049897	1.38E-10	1.22E-10
rs16471	1.58E-09	1.90E-09
rs4238833	5.71E-08	4.12E-08
rs4785763	1.01E-08	8.56E-08
rs7188458	2.81E-08	5.80E-07
