# Supplementary figures and images for: Challenges in conducting genome-wide association studies in highly admixed multi-ethnic populations: the Generation R Study
Source: Eur J Epidemiol. 2015 Mar 12;30(4):317–30. doi: 10.1007/s10654-015-9998-4 (PMC4385148; doi:10.1007/s10654-015-9998-4)

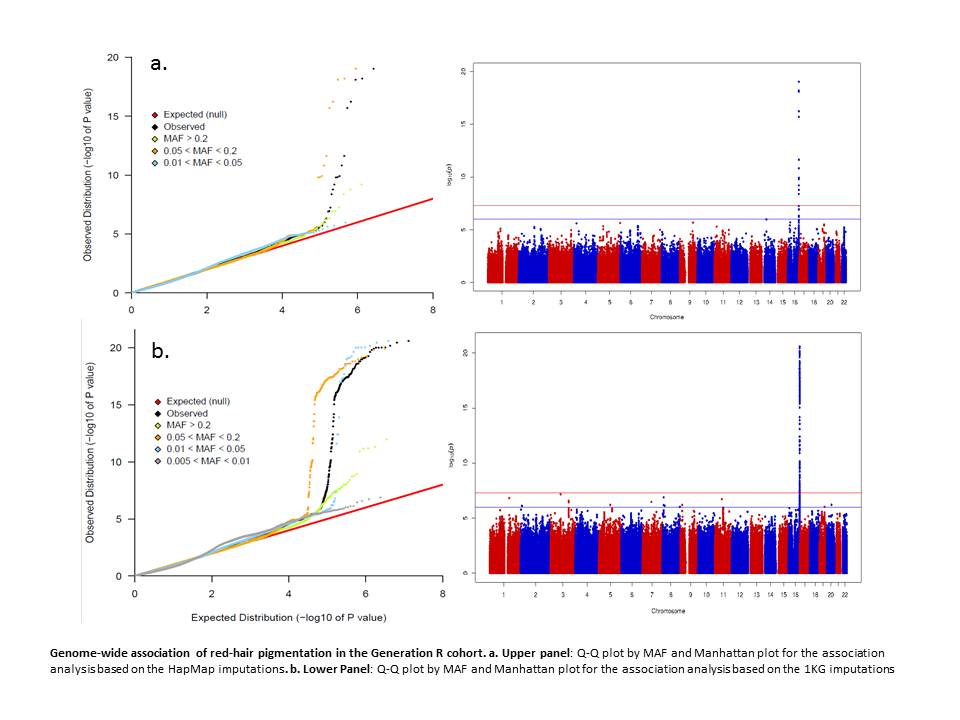

Supplement: Supplementary file 12 — Supplementary material 12 (TIFF 171 kb) [file 10654_2015_9998_MOESM12_ESM.tif]
